# Adolescent Emoji Use in Text-Based Messaging: Focus Group Study

**DOI:** 10.2196/59640

**Published:** 2025-04-28

**Authors:** Matt Minich, Bradley Kerr, Megan Moreno

**Affiliations:** 1 Department of Pediatrics, School of Medicine and Public Health, University of Wisconsin-Madison, 2870 University Ave, Madison, WI, 53705, United States, 1 9702311823

**Keywords:** communication, text messaging, smartphones, emoji, focus groups, adolescent, teen, youth, teenagers, text, phone, messaging, text communication, emotion

## Abstract

**Background:**

Adolescents increasingly communicate through text-based messaging platforms such as SMS and social media messaging. These are now the dominant platforms for communication between adolescents, and adolescents use them to obtain emotional support from parents and other adults. The absence of nonverbal cues can make it challenging to communicate emotions on these platforms, however, so users rely on emojis to communicate sentiment or imbue messages with emotional tone. While research has investigated the functions of emojis in adult communication, less is known about adolescent emoji use.

**Objective:**

This study sought to understand whether the pragmatic functions of adolescent emoji use resemble those of adults, and to gain insight into the semantic meanings of emojis sent by adolescents.

**Methods:**

Web-based focus groups were conducted with a convenience sample of adolescents, in which participants responded to questions about their use and interpretation of emojis and engaged in unstructured interactions with one another. Two trained coders analyzed transcripts using a constant comparative coding procedure to identify themes in the discussion.

**Results:**

A total of 6 focus groups were conducted with 31 adolescent participants (mean age 16.2, SD 1.5 years). Discussion in the groups generally fell into 4 themes: emojis as humorous or absurd, emokis as insincere or complex expressions of setiment, emojis as straightforward experssions of sentiment, and emojis as having context-dependent meanings. Across themes, participants often described important differences between their own emoji use and emoji use by adults.

**Conclusions:**

Adolescent focus group participants described patterns of emoji use that largely resembled those observed in studies of adults. Like adults, our adolescent participants described emojis’ semantic meanings as being highly flexible and context-dependent. They also described both phatic and emotive functions of emoji use but described both functions in ways that differed from the patterns of emoji use described in adult samples. Adolescents described their phatic emoji use as absurd and described their emotive emoji use as most often sarcastic. These findings suggest that emoji use serves similar pragmatic functions for both adolescents and adults, but that adolescents see their emoji use as more complex than adult emoji use. This has important implications for adults who communicate with adolescents through text-based messaging and for researchers interested in adolescents’ text-based communication.

## Introduction

### Overview

Adolescent communication is increasingly mediated by text-based communication platforms like SMS or social media messaging. About 95% of US adolescents aged 13‐17 years have a smartphone for personal use [1], and adolescents in this age group send and receive an average of 67 text messages each day [2]. Text messaging is now the dominant mode of communication between adolescents [2] and is increasingly important to adolescents’ relationships with parents and other adults [3]. For example, text-based communication is an effective way for healthcare practitioners to reach young or adolescent clients [4] and can help therapists discuss sensitive topics with younger clients [5]. Many parents also surveil the text exchanges of their adolescent children—in a nationwide survey, 64% of parents said they regularly checked the contents of their child’s phone, including text message logs [6]. Thus, many adolescents’ most important relationships are affected in some way by the features of text-based messaging platforms.

One feature that applies to text-based messaging of all kinds is the absence of nonverbal cues, such as body language or tone of voice, that people use to convey emotional context [7]. Users often address this deficit with emojis, which can represent emotional states through pictograms [8]. However, emojis can create confusion because users often interpret them differently [9]. Past research has addressed this confusion both by identifying patterns in emojis’ pragmatic functions [10] and by reducing ambiguity around emojis’ semantic meanings [11,12], but that work has largely relied on samples of adult emoji users. Because emoji use and interpretation differ across age groups [13,14], it is unclear whether the insights from this research can be extended to adolescents. Given the emerging importance of text-based messaging to adolescent communication, this study explores adolescents’ perspectives on their own emoji use. Its aims are twofold: to understand whether the pragmatic functions of adolescent emoji use resemble those of adults and to gain insight into the semantic meanings of emojis sent by adolescents.

### Pragmatic Functions of Adult Emoji Use

The earliest known use of emotive pictograms in computer-mediated communication was by Carnegie Mellon Professor Scott Fahlman, who proposed in 1982 that users of an intradepartmental message board distinguish serious posts from humorous ones by marking them with the symbols :-) or :-( [8]. Though similar symbols (called emoticons) are still used, they have since been largely supplanted by the Unicode emoji library, which gives users access to a set of more than 3600 pictographs that are mostly standardized across devices [15]. The use of emojis is now nearly ubiquitous—more than 5 billion emojis are exchanged each day on Facebook alone [15], and the “face with tears of joy” emoji (😂) was chosen as the Oxford English Dictionary’s Word of the Year in 2015.

When asked about their own emoji use, adults say they use the pictograms both to express emotions directly and to modify the tone of messages. For example, surveyed English-speaking adults said they understand emojis as tools for tone modification, in which the emoji modifies text and clarifies its interpretation [16]. US adults have reported using emojis primarily to express sentiment, to strengthen expressions, or to adjust the tone of messages [17]. Thus, the utility of emojis for adults largely overlaps with that of emoticons, which adults also use to communicate feelings and reduce the ambiguity of text [18]. For emojis and emoticons alike, surveyed adults have said they send pictograms primarily to express their feelings, to strengthen the content of a message, or to imbue a message with an element of comedy or fun [19]. Notably, adults’ reasons for sending emojis differ slightly across age groups, with younger adults reporting a more diverse set of motives (including, for example, a desire to make a message ironic or sarcastic) than older adults [19]. Research has also found that older adults use a less diverse emoji vocabulary than younger adults and are generally less likely to use emojis in text-based communication [20].

Theorists have suggested adult emoji use can be broadly categorized as serving 1 of 2 pragmatic functions: a phatic function or an emotive function [10]. The phatic function of emojis describes their use as a fill-in for “small talk:” semiverbal gestures that maintain connection and keep communication light and friendly but lack specific semantic meanings. For example, a user might send a simple smile emoji (🙂) to open or close a conversation or to fill an uncomfortable period of silence. This aligns with research finding that emojis are usually associated with positive sentiments [21], and that they can be used to facilitate connections in a way that is agnostic to a conversation’s emotional content [22]. The emotive function of emojis describes their use to convey the sender’s emotional state or to imbue a message with emotional context. This may be done by using emojis in place of words describing emotions (for example, by sending 😠 in place of the words “I’m angry”), or by adding emojis to complete statements (eg, “I saw a car accident today 😱”).

### Semantic Ambiguity of Emojis

Though users often send emojis to reduce ambiguity about messages’ emotional context, emojis can create confusion because receivers interpret them differently. For example, when experimental participants were asked to interpret expressive face emojis, they disagreed on even basic sentiment assignments (positive, negative, or neutral) 25% of the time [23]. This confusion persisted even when the emojis were accompanied by relevant text. Similarly, experimental participants tended to rate text messages paired with face emojis as more emotionally ambiguous than messages paired with nonface emojis [21]. When experimenters asked participants to interpret emojis contained within text messages, participants offered a wide variety of interpretations. For example, some participants understood the loudly crying emoji (😭) to signify sadness while others perceived this emoji as sarcastic or exaggerated [24].

Research has shown that the interpretation of emojis can be affected by the ages of the sender and receiver. For example, older adults were found more likely to interpret emojis as having positive meanings [14], and younger Chinese adults were more likely to use “positive emojis” (such as the simple smile 🙂) to express negative sentiments [25]. Older adults have also proven less likely than younger adults to recognize emojis as indicators of sarcasm [26], and more likely to express confusion about emojis or choose literal interpretations of their meaning [13]. Older adults have reported less confidence in their interpretations of emojis and perceptions that emojis are difficult to use correctly [20].

### Understanding Adolescent Emoji Use

Among adults, research shows that both the pragmatic functions and semantic interpretations of emojis differ as a factor of age. Older adults have reported less diverse motivations for using emojis [19] than younger adults, for example, and more difficulty interpreting emojis they receive in messages from others [13,20]. This suggests that adolescent emoji use might differ from adult emoji use in meaningful ways, which can create challenges both for adults seeking to communicate with adolescents through text-based messaging and for researchers interested in text-based communication between adolescents.

Understanding how adolescents use and interpret emojis is particularly important because text-based messaging has emerged as an essential platform for adolescent communication. Text messaging behavior peaks around 11th grade [27] and slows in young adulthood [28], leading some scholars to suggest that the affordances of this medium may make it particularly well-suited for adolescents’ developmental needs. Indeed, the asynchronous and socially distanced nature of text messaging may facilitate adolescents’ desires for autonomy, identity development, and the formation of intimate relationships [27]. Text-based messaging is also increasingly important to communication between adolescents and adults. Adolescents often use these platforms to request emotional support from parents, for example, and have reported improvements in their offline relationships with parents on days when they exchange more text messages with them [3]. Adolescents have expressed desires to use text-based messaging platforms for communication with adults about emotional support [4] and have said text-based platforms provide them with a greater sense of authority and control in interactions with counselors and therapists [29].

Emotive pictographs like emojis have long been used to communicate emotions through text [8], but age-related differences in the function and interpretation of emojis could facilitate miscommunication. This could exacerbate existing problems with communication between adolescents and adults, such as communication between adolescents and their parents. Parents and adolescents often fail to accurately understand one another’s cognitive and affective states even when communicating face-to-face [30,31]. This miscommunication has been described as a failure of empathic accuracy [32], or the accurate prediction of a conversation partner’s thoughts and feelings. Adults often use emojis to improve empathic accuracy [33] in text-based exchanges, but it is unclear whether adolescents use them for a similar purpose. Thus, this study seeks to better understand how adolescents use emojis in these exchanges, and how they understand the semantic meanings of those emojis. Given the persistence of emotive pictographs over decades of computer-mediated communication [8], these insights should remain useful even as communication platforms evolve.

## Methods

### Participants

Adolescent focus group participants were recruited through a node distribution method, in which contacts of the research team (staff at youth-serving organizations and participants in previous studies) used word-of-mouth to inform potential participants about the study. All participants provided assent and parental consent and then completed a demographic survey that assessed participants’ age, gender, race, and ethnicity.

A total of 31 adolescents participated in the focus groups. A total of 48% of participants were female, and the average participant age was 16.2 (SD 1.5) years. Complete demographic information is presented in Table 1 below.

**Table 1.** Participant demographics. Self-reported demographic data of participants in 6 web-based focus groups conducted to identify themes in adolescents’ understandings of their own use of emojis in text-based messaging.

Characteristic	Values, n (%)	
Gender		
Cisgender female	14 (45.2)	
Cisgender male	10 (32.3)	
Non-binary	2 (6.5)	
Transgender female	1 (3.2)	
Transgender male	1 (3.2)	
Other	1 (3.2)	
Did not disclose	2 (6.5)	
Ethnicity		
Non-Hispanic or non-Latino	26 (83.9)	
Hispanic or Latino	3 (9.7)	
Did not disclose	2 (6.5)	
Race		
Caucasian or White	16 (51.6)	
Asian or Asian-American	6 (19.4)	
American Indian or Alaska Native	1 (3.2)	
Black or African-American	1 (3.2)	
Native Hawaiian or other Pacific Islander	0 (0.0)	
Other	0 (0.0)	
More than one race	5 (16.1)	
Did not disclose	2 (6.5)	

### Data Collection Procedure

Adolescent perspectives were collected through 6 web-based focus groups, which allowed participants to express their experiences without restriction and encouraged them to build on one another’s contributions. Participants completed a brief demographic questionnaire before joining the group. Groups were conducted using a digital conference call service by 2 trained facilitators (the first author and second author) and lasted up to 90 minutes. Both facilitators were White males employed at a large midwestern research institution, who both possessed MS degrees at the time of data collection. In focus groups, facilitators initially asked open-ended questions about emoji use and interpretation (eg, “What are some ways you use emojis in your communication?”), as well as specific questions about styles of emoji use (eg, “Do you usually use emojis to replace words or phrases, or to add emphasis?”). Participants were also shown several emojis, including the simple smile (🙂) and 5 randomly selected face emojis (in the iPhone iOS style) asked to describe their interpretations of those emojis and the circumstances in which they might share them.

### Ethical Considerations

The procedure used to collect data for this study was approved by the University of Wisconsin-Madison Health Sciences Institutional Review Board (#2019‐0839), and no adverse events were reported. Informed consent from parents or guardians and informed assent from adolescent participants were obtained through a digitally delivered form before focus group participation. Informed consent documents clarified that deidentified focus group data could be made available to investigators outside the original study team. Privacy and confidentiality of participant data were protected by redacting all identifying information from focus group transcripts before analysis. All documents containing identifiable data (eg, informed consent documents and original focus group recordings) were stored on a secure server only accessible by members of the study team. Participants were compensated US $40 for participation in the study, regardless of whether they completed their focus group participation (no participants chose to leave focus groups early).

### Analytical Approach

Analysis was performed using a constant comparative procedure, in which 2 trained coders applied inductive reasoning to identify themes [34]. Originally developed for use in Grounded Theory methodologies [35], this procedure allows analysts to identify themes from complex qualitative data sets without relying on a priori hypotheses. This approach was appropriate for this study both because of the dearth of past research into adolescent emoji use and because of the relatively unstructured nature of the data, which included not only responses to researchers’ prompts but also unplanned interactions between participants.

After 3 focus groups had been conducted, coders began reviewing focus groups for theoretical saturation: a point at which new ideas or themes no longer emerged from discussions. Coders identified theoretical saturation after the completion of 6 focus groups, signaling the end of data collection and the beginning of thematic analysis.

Thematic analysis was conducted in 3 stages. First, coders independently read transcripts from 2 focus groups, each developing their own draft codebook. The coders then compared and discussed these codebooks, consolidating them into a single codebook that both coders agreed upon. This codebook contained a set of superordinate “branch” codes, each of which encompassed a set of more specific, subordinate “leaf” codes.

In the second stage of thematic analysis, coders refined the codebook by independently coding the same 2 additional transcripts and then meeting to compare results. Coders identified and discussed any discrepancies in their coding of these transcripts, then agreed on codebook revisions to resolve them. When coders agreed that all areas of confusion had been addressed, they divided and independently coded the remaining transcripts, so that each transcript was coded by a single coder. While coding this final set of transcripts, coders held regular meetings to discuss any further need for codebook revisions and to reach an agreement on the coding of any ambiguous statements.

In the third stage of thematic analysis, coders met to review the coded transcripts and identify overarching themes and subthemes. Coders reread excerpts assigned to each superordinate “branch” and subordinate “leaf” code, then independently collapsed these codes into larger “theme” groups. Coders then met to discuss the similarities and differences of the 2 sets of themes, consolidating them into a single set of shared themes. These themes were then reviewed by an independent youth advisory board consisting of 6 adolescents, who affirmed these themes as a reasonable summary of transcript content.

Finally, after thematic analysis was complete, coders considered how the agreed-upon themes aligned with their a priori areas of theoretical interest: identifying pragmatic functions of emoji use (phatic and emotive) and resolving the semantic ambiguity of emojis.

## Results

### Overview

Coders identified 4 emergent themes in focus group transcripts. One described the phatic function of emojis: “emojis as humorous or absurd”, and 2 described their emotive functions: “emojis as insincere or complex expressions of sentiment and emojis as straightforward expressions of sentiment”. Finally, one theme spoke to the semantic ambiguity of emojis: “emojis as having context-dependent meanings”.

Notably, some statements made by participants were classified as pertaining to more than one theme. The extent of this crossover, as well as the total number of statements pertaining to each theme, is recorded in Table 2 below.

**Table 2.** Distribution of participant statements across themes. A matrix denoting the number of statements made by adolescent focus group participants coded for each of the 4 themes identified during analysis. Intersecting columns and rows represent the number of statements coded as 2 themes (eg, column 1 of row 2 describes the number of statements coded as both theme 1 and theme 2).

	Theme 1	Theme 2	Theme 3	Theme 4
Theme 1	39	6	2	11
Theme 2	6	81	4	11
Theme 3	2	4	57	5
Theme 4	11	11	5	46

### Theme 1: Emojis as Humorous or Absurd

#### Overview

When participants described phatic uses of emojis (ie, use of emojis to maintain connection without giving them semantic meaning), they described doing so in the context of humor or absurdity. In these cases, it was understood that the sender or receiver found humor in the act of using emojis itself.

#### Theme 1a: Emojis as Innately Humorous

Several participants said they understood emojis as being inherently humorous and described sending emojis for the intention only of communicating humor or social connection. The emojis themselves in these communications were either divorced from their original semantic meanings or were assumed to have no semantic meaning. In most cases, participants described the absence of semantic meaning as essential to the emoji’s communicative function.

I think, like there’s one, which is just like, as a red background and like a B [🅱️], and they were, I mean, they’d seemingly like have no meaning. And they still don’t really like, take on that big of a meaning, but they were like the randomness for like, used for, um, like comedic purpose or for like an inside joke kind of thing.

…The sparkle [✨], or the pixie, dust, I think was originally used to like enhance the meaning of something, or like making it aesthetic in some way. But now I’m just using it for random words that it doesn’t really make sense in—but it’s like funny in a way (laughs). It’s kind of like ironically…

…there was like the chair emoji [🪑], which like people would just use it to be funny and like the point of it was that it had no meaning, but like it was just supposed to be like a random emoji with no meaning.

#### Theme 1b: Emoji Spam

Participants also described engaging in a specific pattern of absurd emoji use called “Emoji spamming,” in which they sent long strings of randomly selected emojis. Again, participants described these strings as not only lacking any specific semantic meaning but described their communicative function as being innately tied to their absence of meaning.

…Emoji spamming often just signifies, like, chaos. Like, I think it’s often used to, like, maybe you saw something outside and you wanna say something about it sarcastically, so you take a picture of it... Maybe, like, let’s say you take a picture of a car crash, then you’re like, “Oh, this is such a pretty car,” and then you emoji spam. It’s just, like, used to be sarcastic and funny.

…I know definitely some of my friends do this, like, if they’re typically experiencing, like, a negative emotion, they’ll like emoji spam and do a bunch of random, like, really random ones…

…People, like, sometimes, like, spam emojis because they’re, like, angry that they lost [a mobile game] or they’re excited that they won. I really think it goes both ways…

### Theme 2: Emojis as Insincere or Complex Expressions of Sentiment

#### Overview

Participants tended to describe emojis as inappropriate for communicating sincere emotions and mood, though some did acknowledge using them for this purpose (see theme 3). Rejection of sincere emoji use was generally framed as (1) a broad aversion to the use of emojis, (2) a preference for the ironic or sarcastic use of emojis, or (3) the use of apparently positive emojis to communicate negative emotions or moods.

#### Theme 2a: Aversion to the Use of Emojis

Many participants said they did not use emojis frequently, and they often specified that they were less likely to use emojis to communicate negative emotions or in emotionally negative situations, when they described emojis as socially inappropriate:

…When I’m, like, in a serious conversation I don’t usually use emojis because I feel, like, it’s not as serious if I were to use emojis.

I don’t really use emojis all that much. So if I am feeling those [negative] emotions, I’ll probably just verbalize and like text them instead of using emojis for it, since it’s kind of probably more of a serious conversation.

#### Theme 2b: Preference for Ironic or Sarcastic Use of Emojis

Many participants said they typically used emojis in ironic or sarcastic ways so that their intended meaning was the opposite of a literal interpretation. For example, participants might use a laughing emoji to communicate that a joke had failed to land or might use a “cool” emoji [😎] to lampoon a person or message:

..With the sunglasses, I kind of use that sarcastically, kind of, like, saying, “Oh, I’m so cool I did that.” Like, “Oh, that’s so cool I’m doing something.”

I am one of those people that use this emoji [🙂] sarcastically because, like, I feel like if I wanted to show that I was happy through emoji I would put, like, the closed eye smiling [😊] or something but this one seems, like, kind of creepy when you look at it so I usually use it sarcastically.

…Just people in general, like, they’ll use an emoji and me and my friends will, like, use that emoji [😎] to make fun of the people who use them, like, genuinely. Like [we’ll] use it as, like, satire I guess you could say because we think it’s funny that people actually use them.

Mentions of ironic/sarcastic emoji use were notably more frequent in most focus groups than mentions of sincere emoji use.

#### Theme 2c: Communicating Negative Moods With Positive Emoji

When prompted to describe their use of traditionally positive emojis, participants often said they used these emojis to communicate negative emotions. Participants described positive emojis as adding layers of irony or insincerity to negative emotions, or to communicate a reluctant acceptance of negative circumstances.

I don’t know if ironic is the right word, but I would use it [😀] in the opposite sense. Like, you’re smiling through the pain or something. I know it’s terrible, but that’s just how I would see them.

a lot of times, you could use that emoji [🙂] as, like, I don’t know, like, you’re smiling through the pain, or, like, used, like, sarcastically, saying, like, “Oh, I’m so happy I messed up on something.”

I guess non-adolescents that don’t really like use emojis ironically would definitely think that like [😀] is extremely like happy and positive. But, I mean, really, it’s not. It’s pretty lifeless.

I feel like, it also goes with like a sarcastic tone too in kind of a joking manner. Like I texted my best friend last night and I was like “It’s 5:00a.m. and I’m awake 🙂”

### Theme 3: Emojis as Straightforward Expressions of Sentiment

Though participants generally agreed that adults were more likely to use emojis to communicate sincere emotions, some described instances where they used emojis without irony or humor. In many such cases, they described the emoji as referring to a nameless, specific circumstance or emotional state:

Maybe I, I dropped my like pencil in the hallway and I had to like pick it up, like I would use that emoji [😓] like being like, “Oh yeah, so stressed about it.”

I’ll probably just use like an emoji, like a heart or something, or a smiley. Or like something like that to just, you know, convey the message that, you know, it’s how I’m feeling.

I use emojis when I don’t want to write out like everything. Like if I think something’s funny I can just do like a laughing emoji [😂] and yeah, it’s just easier to get the point across without having to write.

### Theme 4: Emojis as Having Context-Dependent Meanings

Finally, participants described emojis as having flexible meanings that shifted to suit the audience or conversational context. They cautioned against assigning specific meanings to emojis and said that they anticipated audience responses when choosing emojis.

... For the most part, there’s no real universal meaning for most emojis. Like, I’d say there’s a few that, like, most people always use to signify a few things, but for the most part, they all mean different things for different people in different scenarios.

You have to think about and interpret how another person is going to receive that emoji. And so, when you are with your friends, or are texting your friends and you’re using the emojis like ironically, you know that they’re going to interpret those emojis ironically. But like with adults, I feel like, um, especially with adolescents, we, um, you know—that they’re not going to interpret them the same way we do, so we take it more literally.

Many participants stressed differences between adult and adolescent styles of emoji use, usually emphasizing how these groups differ in their preferences for sincerity or authenticity. Specifically, participants said they were more likely to use sarcasm or humor when sending emojis to peers, while they expected adults to interpret any emoji as a sincere expression of sentiment.

…Speaking of like older generations who use, who use like messaging or whatever, I like use the emojis in a different way than I do with my friends. Like with older people, I’ll just use them in like an actual way of what they’re like theoretically supposed to mean. Like a smiley face will actually mean a smiley face…

Participants also said that certain emojis held meanings within their social groups that they knew were not shared by other groups. Though this was sometimes described as extending to entire geographic regions, these meanings were most often described as specific to groups of friends:

Almost everyone has different emojis that mean something to them with, like, another friend, like, an inside joke or, like, another meaning.

## Discussion

### Principal Findings

This focus group study explored the ways adolescents use and interpret emojis, which has received less scholarly attention than adults’ emoji use. By collecting data through focus groups and applying a coding strategy informed by Grounded Theory [35], this work was the first to our knowledge to examine adolescent emoji use through adolescents’ own words. The 4 themes that emerged in our analysis suggest that adolescent emoji use has many similarities with adult use but that it differs in some important ways. First, adolescents described the phatic functions of emojis that were informed by a sense of humor or absurdity (theme 1). Second, adolescents confirmed the emotive function of emojis, which they described as sometimes being achieved through a layer of sarcasm or irony (themes 2 and 3). Finally, they described emojis as having highly variable semantic meanings that are often understood differently by adolescents and adults (theme 4). Taken together, these findings suggest adolescents and adults use emojis for similar reasons (ie, phatic and emotive uses), but that adolescent emoji use is informed by humor, irony, and social dynamics that may make these messages difficult for adults to interpret.

### Adolescents’ Phatic Emoji Use is Humorous and Absurd

Focus group participants described emoji uses that seem to take advantage of emojis’ phatic functions (ie, to maintain connection without providing any specific semantic meaning), but their use of this function was distinct from phatic use by adults. For example, adolescents said they frequently sent emojis that were meant to have no semantic meaning at all (theme 1a) or sent strings of randomly selected “emoji spam,” or single emojis with no meaning outside of their repeated use (theme 1b). These phatic uses are notably different from the phatic uses observed in adult messaging [10], which include opening a conversation with a simple smile (🙂) or closing a conversation with a wave (👋). Still, they fulfill the core phatic function of emojis: shoring up social connections without contributing substantial content.

### Adolescents’ Emotive Emoji Use is Often Sarcastic

Adolescents also described patterns of emoji use consistent with the emotive functions of emojis, but these patterns were informed by a preference for humor and a complex understanding of emojis’ semantic meanings. For example, adolescents said they preferred not to use emojis in text communication (theme 2a) and were particularly averse to using emojis to express negative emotions. This aligns adolescent use with some research on adult use, which has found that adults tend to associate emoji use with expressions of more positive emotions [21] and sometimes see emojis as inappropriate in negative emotional contexts [36]. Though adolescents said they did sometimes use emojis to communicate sincere emotions (theme 3), much of the content from our focus groups suggested that adolescents understand the emotive functions of emojis differently than adults. While adults often use emojis to reduce emotional ambiguity [33] or to complement the emotional valence of text messages [18], adolescents reported that they usually sent emojis as sarcastic or ironic expressions of emotions (theme 2b) and used positive emojis to communicate negative emotions (theme 2c). Adolescents did not describe this sarcastic use of emojis as a source of confusion in communication with peers, however—instead such use was widely understood to communicate emotions in a sardonic or self-effacing tone. Thus, our results suggest adolescents also use emojis to reduce emotional ambiguity, but that they do so with the assumption that others can discern between straightforward and sarcastic expressions.

### Adolescents Believe Adults Use Emojis Differently

Our overall interpretation of these findings—that adolescents use emojis for similar reasons as adults but perceive their use as more nuanced—is further supported by the fact that adolescents understood their own emoji use as different from use by adults (theme 4). Importantly, our focus group participants understood adults as favoring literal interpretations of emojis, which they often find risible (theme 2b). Participants said they anticipate these differences when communicating with adults, often suggesting adults would misunderstand the emojis they shared in peer-to-peer communication. Importantly, participants never said they were confused by the emoji choices made by adults. This suggests that adolescents do not understand adult emoji use as its own complex style of communication but instead view it as less developed than their own use.

Thus, adolescents in our focus group believed their use of emojis in their peer-to-peer communication was at least partially inaccessible to their parents and other adults. This might suggest that adolescents use emojis to facilitate one of the core “tasks” of their development: establishing autonomy from parents [37]. Coconstruction theory suggests that adolescents use digital media to achieve developmental tasks [38], and past research suggests that adolescents might gravitate to text messaging and other forms of text-based communication because these platforms help them develop relationships with peers while establishing independence and autonomy [27]. The apparent complexity of adolescent emoji use might serve a similar purpose—just as adolescents have long used slang, fashion, and other cultural codes to distinguish themselves from adults in physical spaces, they may use novel styles of emoji use to assert autonomy within text-based digital spaces.

### Limitations

Study findings are limited in 3 important ways. First, while our purposeful sampling procedure recruited a diverse sample of focus group participants, it is likely that important subpopulations were not included in our sample. Thus, it may be that adolescents of certain demographic groups or from certain regions use emojis differently than the participants in this study. Second, the insights of this study depend on self-reflection by focus group participants who may not have been aware of or may have chosen not to disclose certain important aspects of their emoji use. Observation of real-world adolescent emoji use might reveal different patterns. Third, this study’s comparisons between adolescent and adult emoji use were seen strictly from the perspective of adolescents because this study did not assess adults’ motivations for emoji use. Future work could make stronger comparisons by assessing the emoji use of both adolescents and adults.

### Practical Implications

These findings support the assertion [10] that emojis largely fill both emotive and phatic functions in interpersonal communication and suggest that adolescents see their emoji choices as informed by a complex set of shared norms they believe are inaccessible to adults. In addition to their contribution to the theoretical understanding of adolescent emoji use, these findings also have implications for parents, who increasingly interact with adolescents through text-based platforms [3] and sometimes observe adolescents’ text-based communications with peers [6]. These results suggest that parents should exercise caution when interpreting emojis sent by adolescents. While adolescents did say they often adjust their emoji use when communicating with adults, this may not be the case when other adolescents are also involved in the conversation (such as in a group chat), or if adolescents do not have the energy or motivation to adjust their habits for clarity of communication. These results also suggest that adults are likely to misinterpret the emojis used in adolescent-to-adolescent communication, which is an important consideration for parents, school administrators, or practitioners who may find themselves reading such conversations.
